# Exploring the effects of 3D-360°VR and 2D viewing modes on gaze behavior, head excursion, and workload during a boxing specific anticipation task

**DOI:** 10.3389/fpsyg.2023.1235984

**Published:** 2023-08-23

**Authors:** Mildred Loiseau Taupin, Thomas Romeas, Lauryn Juste, David R. Labbé

**Affiliations:** ^1^Laboratoire de recherche en imagerie et orthopédie, École de technologie supérieure, Montréal, QC, Canada; ^2^Centre de recherche du Centre hospitalier de l’Université de Montréal, Montréal, QC, Canada; ^3^Institut national du sport du Québec, Montréal, QC, Canada; ^4^École d’optométrie, Université de Montréal, Montréal, QC, Canada

**Keywords:** visual strategies, perceptual-cognitive skills, perception-action, boxing, anticipation, virtual reality, workload, presence

## Abstract

**Introduction:**

Recent evidence has started to demonstrate that 360°VR, a type of VR that immerses a user within a 360° video, has advantages over two-dimensional (2D) video displays in the context of perceptual-cognitive evaluation and training. However, there is currently a lack of empirical evidence to explain how perceptual-cognitive strategies differ between these two paradigms when performing sports-related tasks. Thus, the objective of this study was to examine and compare the impact of different viewing conditions (e.g., 3D-360°VR and 2D video displays), on gaze behavior and head excursions in a boxing-specific anticipatory task. A secondary objective was to assess the workload associated with each viewing mode, including the level of presence experienced. Thirdly, an exploratory analysis was conducted to evaluate any potential sex differences.

**Methods:**

Thirty-two novice participants (16 females) were recruited for this study. A total of 24 single-punch sequences were randomly presented using a standalone VR headset (Pico Neo 3 Pro Eye), with two different viewing modes: 3D-360°VR and 2D. Participants were instructed to respond to the punches with appropriate motor actions, aiming to avoid punches. Gaze behavior was recorded using a Tobii eyetracker embedded in the VR headset. Workload and presence were measured with the SIM-TLX questionnaire. Fixation duration, number of fixations, saccades, search rate and head excursions (roll, pitch, yaw) were analyzed using linear mixed models.

**Results:**

The results revealed significant shorter fixation durations and more head excursions (roll, pitch) in 3D-360°VR, compared to the 2D viewing mode (*p*s < 0.05). The sense of presence was found to be much higher in the 3D-360°VR viewing mode (*p* < 0.05). No sex differences were observed. These results demonstrate that 360°VR elicited shorter fixation durations but mostly greater head excursions and immersion compared to a 2D projection in the context of a boxing-specific task.

**Discussion:**

These findings contribute to the understanding of previous evidence supporting the possible advantages of using 360°VR over 2D for perceptual-cognitive evaluation and training purposes. Further validation studies that compare behaviors and performance in 360°VR with those in the real-world will be needed.

## 1. Introduction

In sports, perceptual cognitive skills such as the ability to seek and select relevant information, to use knowledge and to select and execute the appropriate responses are essential (Williams et al., 1999; Mann et al., 2007). A number of attempts have been made to create standardized assessments and training applications for perceptual-cognitive skills. Varying degrees of success were obtained due to the challenges involved in replicating the representativeness of the sporting environment and simulating embodied decision-making (Broadbent et al., 2015b; Fadde and Zaichkowsky, 2018). For example, video-based tests have been widely used to study anticipation and decision making in several sports (Farrow and Abernethy, 2002; Fadde, 2006; Hopwood et al., 2011; Broadbent et al., 2015a; Müller et al., 2019; Zhao et al., 2022), including combat sports (Russo and Ottoboni, 2019).

Most often, researchers have employed an occlusion paradigm wherein two-dimensional (2D) sport-specific footage of an action or opponent is presented to the viewer. During these tests, the viewer is required to anticipate or make the most appropriate decision regarding the upcoming event when the occlusion occurs [e.g., through a blacked screen or pause, Farrow et al. (2005)]. These tests have been mostly displayed on computer or sometimes life-sized screens and performance has been quantified using measures of information pick-up (e.g., visual strategies), accuracy or response time (Mallek, 2019). Although this occlusion paradigm has been successful in distinguishing expert athletes from novices (Mann et al., 2007), certain limitations have been identified in its ability to differentiate or enhance perceptual-cognitive skills among athletes with more similar levels of expertise. For example, Dong et al. (2023) expressed concerns regarding the validity and reliability of a video-based test employed to assess decision making in water polo, highlighting the nonrepresentative nature of the paradigm, which lacks adequate action correspondence (such as perception-action coupling) to the natural task.

Indeed, Murr et al. (2021) found that a video-based test involving a specific motor response was better at distinguishing different levels of expertise. More broadly, *in situ* tasks that require a specific response have proven to be more effective in discriminating between skill levels (Travassos et al., 2013). This emphasizes that athletes act and react within the constraints of their physical body and of their environment, as they generate embodied choices (Raab and Araújo, 2019). As such, a paradigm which seeks to evaluate or train perceptual-cognitive skills should incorporate practice designs that are more representative, encompassing perceptual, cognitive, and motor processes, while maintaining perception-action coupling (Pinder et al., 2011; Voigt et al., 2023). A framework proposed by Hadlow et al. (2018) pointed out three main areas to optimize perceptual-cognitive test designs: stimulus correspondence, action correspondence, and targeted perceptual function. For example, this framework suggests that a task can be improved by increasing its correspondence to competition with enhanced physical representativeness (e.g., representation of opponent kinematics vs. abstract cues), by encouraging the reproduction of corresponding real-world movements (e.g., avoidance movement vs. mouse click), and by involving similar perceptual-cognitive processes (e.g., visual search strategies vs. contrast sensitivity).

Virtual reality (VR) is a technology that has demonstrated promising potential in creating more ecologically valid conditions for perceptual-cognitive applications (Gray, 2019). In contrast to conventional 2D video-based tests, VR allows for the creation of immersive, sport-specific representations that maintain a high level of physical fidelity (e.g., stimulus correspondence), embodiment, and action fidelity (e.g., perception-action coupling). While the latest-generation of head-mounted displays incorporates features such as accelerometers for motion capture and analysis, VR technology further enables more advanced and standardized skills evaluation. Moreover, the inclusion of an eyetracker allows for the interpretation and analysis of gaze behavior. VR also offers users a 360° point of view for scanning, as well as stereoscopic conditions, among other benefits, while also presenting some limitations such as no haptic feedback, acceptance, motion sickness or increased workload (Düking et al., 2018; Akbaş et al., 2019; Kittel et al., 2020; Harris et al., 2020a; Le Noury et al., 2022). In particular, immersive 360° VR (360°VR) is a variant of VR that utilizes 360° videos that are captured in the real world, offering a high level of physical representativeness for sport environments due to the photorealistic nature of the footage, a 360° point of view and, sometimes, stereoscopic vision (Bird, 2020).

Compared to traditional VR (computer-generated simulation), this technology is much more affordable in terms of cost and programming skills required, making it appealing for sport practitioners. Therefore, immersive videos (e.g., 360°VR) has recently been proposed as an upgraded version of traditional videos (e.g., 2D video-based tests) for perceptual-cognitive skill evaluation and training in sports. For example, it has been used for evaluating anticipatory judgement in cricket (Discombe et al., 2022) and water polo (Richard et al., 2021), as well as for evaluating or training decision making in basketball (Panchuk et al., 2018; Pagé et al., 2019), boxing (Romeas et al., 2022), soccer (Fortes et al., 2021; Höner et al., 2023), and umpiring (Kittel et al., 2019). Notably, Kittel et al. (2019) confirmed the validity and reliability of 360°VR for assessing decision making in Australian umpires, and demonstrated that 360°VR offered greater ecological validity when compared to conventional 2D video-based tests. While positive outcomes have generally been reported in athletes using 360°VR technology for perceptual-cognitive assessment and training (Kittel et al., 2019; Pagé et al., 2019; Fortes et al., 2021; Richard et al., 2021; Discombe et al., 2022; Romeas et al., 2022; Höner et al., 2023), it is important to note that one study (Panchuk et al., 2018) reported null findings, which may be attributed to the lack of exact matching between the required response and real-world conditions. Furthermore, the research on this topic is still limited, highlighting the need for further validation studies to gain a better understanding of the true impact of 360°VR compared to traditional 2D video-based tests. Interestingly, none of the previous studies looked at sex differences using this technology, and even worse, only a few of them included female participants which echoes the general under-representation of female participants in sport science (Anderson et al., 2023).

Existing evidence has shown that 360°VR may offer more advantages than 2D video-based tests concerning perceptual-cognitive evaluation and training (Yunchao et al., 2023), however, the specific mechanisms through which 360°VR produces these effects compared to 2D displays remain unclear and require further investigation. One hypothesis is that 360°VR creates a more ecological representation of the real-world, offering more immersive conditions (e.g., sense of presence) and perceptual features (e.g., 360°, 3D) which in turn facilitates the utilization of similar visual search strategies as those employed in real-world scenarios. In fact, one key component of anticipatory and decision-making strategies is gaze behavior. It has been well demonstrated that being able to selectively allocate attention toward important task-related information is a crucial skill of expert performers (Mann et al., 2007; Dicks et al., 2010; Brams et al., 2019). Therefore, it is plausible that the distinct characteristics of 360°VR and 2D displays (e.g., visual field of view, stereoscopy, and sense of presence) contribute to differential effects on visual strategies, with 360°VR being a better proxy for replicating naturalistic gaze behaviors. Previously, Hohmann et al. (2016) found that a 3D video-based test, when compared to a 2D video-based test, exhibited greater effectiveness in improving decision time in handball. The authors highlighted the importance of higher fidelity and improved binocular depth information provided by the 3D format. In a study involving a non-athletic population, Barrett et al. (2022) found no significant differences in task learning accuracy between VR, 3D, and 2D viewing conditions. However, they did observe distinct gaze behaviors in the VR condition, including a higher number of fixations compared to the other viewing conditions. In soccer athletes, Fortes et al. (2021) demonstrated that 360°VR decision-making training led to greater on-field improvements in decision making, associated with greater visual search behaviors (e.g., higher number of fixation and shorter fixations durations), when compared to 2D video-based training. This suggested greater on-field exploration following the 360, which allows a 360° field of view VR training, despite that direct causal relationship between the training and the transfer task was not assessed. In addition to gaze behavior, one area that has been poorly explored in VR but has shown more extensive evidence in field studies is head exploration (or “scanning” in soccer; see Jordet et al., 2013 or McGuckian et al., 2018). Considering the significance of head movement metrics in real-world conditions, analyzing head activity in controlled environments which allow a 360° field of view, such as VR, could provide valuable insights for perceptual-cognitive skills evaluation (Wirth et al., 2021). Although previous studies have observed behavioral differences resulting from the use of VR and 2D viewing modes, there is still a lack of direct evidence assessing the disparities in visual strategies when performing sporting tasks projected on such displays. Further research and validation are needed to better understand these differences (Müller et al., 2022) and to improve the knowledge about potential disparities between sexes (Cowley et al., 2021). To our knowledge, only Tang et al. (2023) found differences in visual search strategies between sexes in a mental rotation task using VR. Most studies on 360°VR have been conducted only in male participants, with conclusions mostly applicable to this group. Transposing conclusion based solely on one group can lead to generalization of results and misinterpretation.

The present study is based on previous work which sought to better understand how perceptual-cognitive skills can be improved in boxing (Romeas et al., 2022). The evaluation and training of in-ring anticipation and decision-making skills in boxers pose significant challenges due to difficulties in maintaining task standardization and the inherent risks of injury to the neck, head, and upper limbs during training sessions (Di Virgilio et al., 2019; Alevras et al., 2022). As such, VR-based solutions would represent a safe alternative to supplement perceptual-cognitive assessment and training in boxing. The primary goal of this study is to compare the impact of different viewing conditions, specifically 3D-360°VR (immersive videos) and 2D video (traditional videos) displays, on gaze behavior and head excursions during a boxing-specific anticipatory task, serving as an initial step towards validating the use of 360°VR solutions. A secondary objective was to evaluate the workload and the sense of presence induced by each of the viewing modes. Indeed, the workload reflects the demands of the task and depends on task conditions (Harris et al., 2020c). Workload is an important factor involved in learning processes and needs to be better understood across learning environment such as virtual simulation (Harris et al., 2020c). We hypothesized that the viewing mode would significantly affect gaze behavior and head excursions, with the 3D-360°VR condition inducing more exploration which implies more fixations and saccades (H1a) and more head excursions, especially in yaw axis, side-to-side (H1b). We further hypothesized that the viewing mode would significantly impact the workload and sense of presence with the 3D-360°VR condition expected to generate higher levels of workload (H2a) and presence (H2b). Finally, considering the underrepresentation of female participants in sport science (Cowley et al., 2021), we conducted an exploratory analysis to assess the presence of any potential sex differences.

## 2. Methods

### 2.1. Participants

Prior to the recruitment process, a power analysis for mixed models was performed using a web-based application developed by Westfall et al. (2014). In mixed models, the sample size depends on the number of participants and the number of stimuli presented to each participant. The design parameters were the effect size (analogous to Cohen’s d, set at 0.5, medium), the α value (set at 0.05), the statistical power (set at 0.8), and the number of stimuli (set at 24). Based on the power analysis, it was determined that a minimum of 31 participants were required for the study. Thirty-two novice participants (16 females, M_age_ ± SD = 26.3 ± 5.5 years) volunteered for this study. Inclusion criteria were to: have no prior experience in boxing, be over 18 years of age, and have normal or corrected-to-normal vision. Contact lenses were allowed but eyeglasses were not because of incompatibility with the VR headset. Initially, participants were provided information about the study procedure, followed by a screening process to determine their eligibility. All participants provided verbal and written informed consent to participate in this study. The study was approved by the Research Ethical Committee (H20220103) of École de technologie supérieure of Montreal.

### 2.2. Viewing conditions

The Neo 3 Pro Eye VR headset (Pico, Pico Immersive, Singapore) was used to present the visual conditions and its two controllers were used for interaction with the graphical user interface between conditions. A custom-made application designed in Unity 2021 (Unity Technologies, San Francisco, United States) was used to display videos of boxing punches in the two viewing modes: on a virtual 2D screen (traditional videos) and in immersive 3D-360° (immersive videos). In the 2D viewing condition, the videos were presented on a screen resembling an 85″ flat screen (1.9 × 1.0 m), positioned virtually at a distance of 3.50 m from the participant, and covering a visual angle of 30.4°. In the 3D-360°VR viewing condition, immersive panoramic videos were displayed, providing a 360° visual experience with a 98° horizontal field of view. These videos were recorded from four boxers (2 females and 2 males) classified as Elite/International level (Tier 4), according to the Participant Classification Framework (McKay et al., 2022). These boxers did not participate in the rest of the study. Four types of punches (straight, jab, uppercut, and hook) were performed in isolation, in front of an Insta 360 Pro 2 camera (Shenzhen, China) that recorded in 3D and 360°. Each type of punch was performed 5 times per boxer. The acquired videos were then processed to meet the desired presentation formats of two viewing modes (2D and 3D-360°) using Insta 360 Stitcher (version 3.0.0, Shenzhen, China) and Adobe Premiere Pro (version 23.0.0, Adobe Inc., San Jose, California, United States). For the 2D condition, the videos were cropped, keeping the boxer’s entire body visible and centered in the image. As a result, 80 videos of single punches were obtained (mean duration was 3.6 s) in each of the 2D and 3D-360°VR conditions.

### 2.3. Experimental procedure

**Figure 1:**
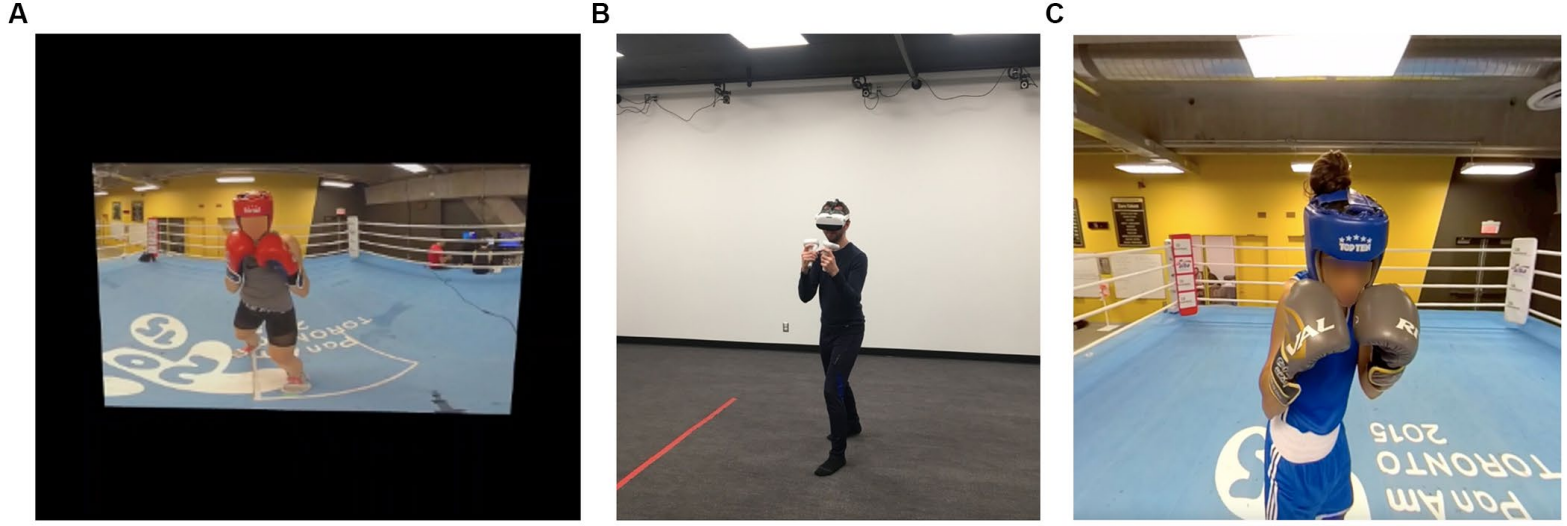
Illustration of the experimental setup **(A)** the 2D-video condition, **(B)** a participant wearing the VR headset and controllers, and **(C)** the 3D-360°VR condition.

In a quiet room (size >2 × 2 m), the experimenter helped each participant to install the VR headset, the heart rate monitor, and to grasp the controllers (Figure 1B). Prior to each experiment, a 10-s calibration was conducted using the built-in calibration method of the Tobii eyetracker, which involved presenting five targets. Then, videos of boxing punches were presented in the headset in the two different viewing conditions: 2D (traditional videos) and 3D-360°VR (immersive videos; Figures 1A,C). All participants were exposed to both viewing conditions. The order of presentation was counterbalanced: 16 participants began the experiment with the 2D condition, and 16 participants began with the 3D-360°VR condition. For each condition, three familiarization videos followed by 24 test videos were presented to the participants. For each condition and for each participant, the presentation of 24 videos from the initial 80 videos dataset was pseudo-randomized so that all participants viewed six videos of each type of punch. Videos presented during the familiarization phase were not presented again during the test phase.

**Figure 2:**
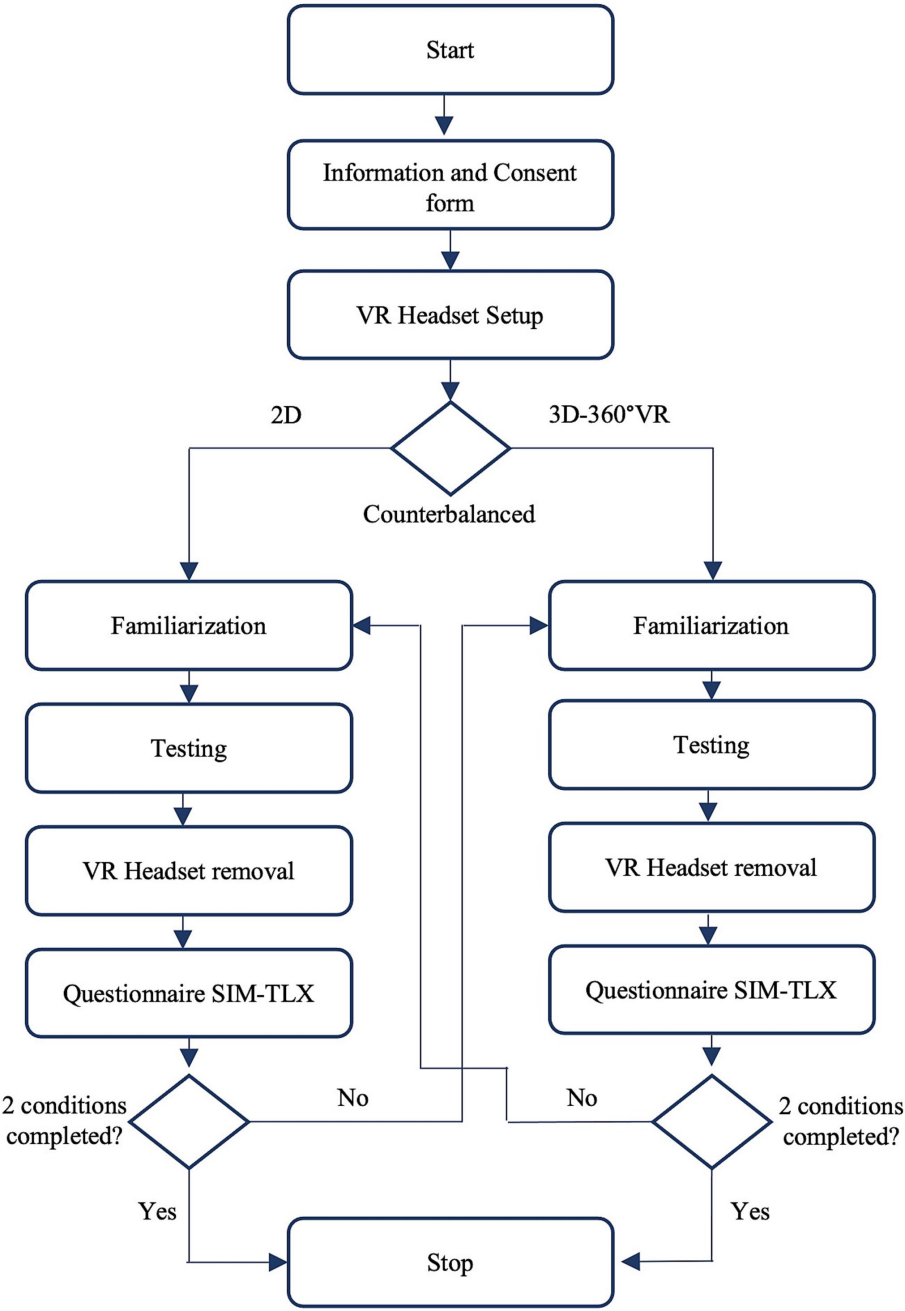
Experimentation design for the present investigation. The duration of the experimentation was approximately 45 min. Questionnaire SIM-TLX was the Simulation Task Load Index questionnaire (Harris et al., 2020a,b,c).

The participants were instructed to avoid punches by generating a naturalistic motor response. Participants were told they could perform lateral movements, move backward, and duck; however, they were instructed not to block the virtual punches. These instructions were formulated to emulate the defensive movements of avoidance observed in boxing and to maintain the perception-action coupling inherent in this anticipatory task. In cases where participants did not adequately respond to the instructions by appropriately moving in response to a video, the experimenter included an additional video trial to ensure sufficient data for analysis (no additional video trial added). To facilitate participant guidance and ensure the correct execution of the experiment, the experimenter utilized a computer displaying a screencast of the VR headset. At the end of each condition, each participant completed the Simulation Workload Assessment (SIM-TLX) questionnaire on paper to assess their workload experience in VR (Harris et al., 2020c). The duration of the experimentation was approximately 45 min (Figure 2).

### 2.4. Measures

#### 2.4.1. Gaze behavior

A Tobii eye tracker (Tobii, Sweden) integrated into the VR headset was used to record the gaze behavior of each participant (accuracy: sub-degree; frequency: 90 Hz). From the Pico Unity XR SDK (version 2.1.3), PXR_EyeTracking was used to obtain the gaze coordinates. The movement velocity was calculated, and two types of gaze behavior were analyzed. A threshold for saccadic movements was set at five times the median speed, and duration thresholds were set for saccades (duration of 50 ms or less) and fixations (duration greater than 100 ms; Holmqvist and Andersson, 2017). The number of saccades and fixations were normalized according to the duration of each video, using RStudio (RStudio Version 2022.12.0, R Foundation, Vienna, Austria). Four variables were calculated for each participant: the average number of fixations, the average fixation duration, the search rate, and the average number of saccades. The search rate was calculated by dividing the total number of fixations by the total duration of fixations. Moreover, coordinates of headset rotations were extracted using Pvr Unity SDK Manager to assess head exploration behaviors (e.g., excursion, McGuckian et al., 2018). Then, movement angles were calculated and normalized according to the duration of each video to obtain head excursion movements according to roll (tilting clockwise or counterclockwise), pitch (tilting forward or backward) and yaw (turning to the left or right) orientations.

#### 2.4.2. Simulation workload measures

The Simulation Task Load Index (SIM-TLX) questionnaire was used to control for the workload generated by each condition and was completed by all participants after each of the 2D and 3D-360°VR conditions (Harris et al., 2020c). This questionnaire, adapted from the NASA Task Load Index (Hart and Staveland, 1988), measures 10 variables related to performing a task in a virtual simulation: mental demand, physical demand, temporal demand, frustration, task complexity, situational stress, distraction, perceptual constraints, task performance and sense of presence (e.g., feeling of: being there,” Mestre et al., 2006). For each of the 10 variables, the participants assigned ratings based on their perceived experience during the task by placing a cross on a visual analogue scale from 0 to 20. These ratings were assigned following each condition.

#### 2.4.3. External load

Participants wore a Polar H10 heart rate monitor that was connected to the Polar Beat application (version 3.5.5) running on a smartphone to record their heart rate during the experimentation (Polar, Finland). Polar Flow (version 4.011) was used to calculate the mean heart rate for each participant, in each viewing condition, serving as a control variable for exercise intensity.

### 2.5. Statistical analysis

Data were analyzed using RStudio Version 2022.12.0. Linear mixed models were used to test our hypotheses using the lmerTest package (Bates et al., 2015) according to the advantages (e.g., use of categorial and continuous variables; possibility of missing data) suggested by Kliegl (2010). In all models, participants were set as a random effect factor. For exploratory analysis, the factor of Sex (Female, Male) was included. First, to test the hypothesis that gaze behavior was different between the two viewing conditions, measures of gaze behavior were set as dependent variables, while viewing condition (2D, 3D-360°VR) and sex (Female, Male) were set as fixed effect factors. The measures of gaze behavior were fixations (number, duration, and search rate) and number of saccades. The same linear mixed models were applied for the analysis of head excursion, with roll, pitch and yaw as dependent variables.

Secondly, to test the hypothesis that workload and sense of presence were different between the two viewing conditions, the 10 simulation workload variables were set as dependent variables, and Viewing condition (2D, 3D-360°VR) and Sex (Female, Male) were set as fixed effect factors. Finally, to control for the external load generated by the stimulation, the heart rate was set as a dependent variable, and the Viewing condition (2D, 3D-360°VR) and Sex (Female, Male) were set as fixed effect factors.

For all inferential analyses, significance threshold was set at 0.05. To identify the effects of the viewing condition and determine the standardized beta effect size, the Report package in RStudio was utilized (Makowski et al., 2023). An effect size of approximately 0.2 was interpreted as small, approximately 0.5 as medium, and approximately 0.8 as large (Cohen, 1988). For all significant mixed linear models, Tukey post-hoc tests were performed.

## 3. Results

### 3.1. Gaze behavior

**Figure 3:**
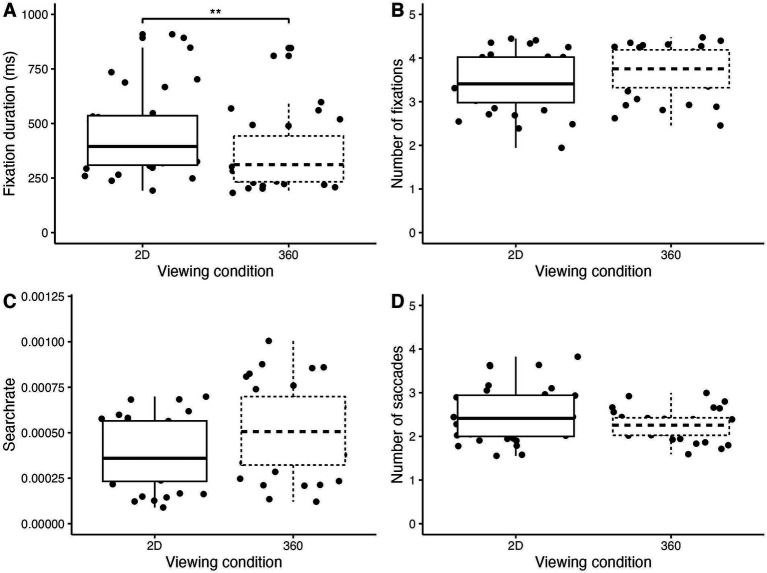
Box plots comparing gaze variables between the two viewing conditions **(A)** Fixation duration (ms), **(B)** Number of fixations, **(C)** Search rate and **(D)** Number of saccades. **p < 0.01.

The linear mixed models revealed no significant interaction between Viewing condition and Sex on any of the gaze variables (*p*s > 0.05). However, linear mixed model revealed medium significant main effect of Viewing condition on the average fixation duration (β = −126.53, 95% CI [−216.58, −36.49], *p* = 0.01; std. beta = −0.63). Indeed, participants fixated for significantly shorter durations in the 3D-360°VR condition (365.8 ms ± 170.1) compared to the 2D condition (457.5 ms ± 198.8, *p* < 0.01). The linear mixed model revealed no significant effects of the Viewing condition on the average number of fixations (*p* = 0.88, d = −0.50) or the number of saccades (*p* = 0.98, d = −4.59e-03) or the search rate (*p* = 1.00, d = 1.52e-03). Indeed, there was no significant difference and very small to moderate effect sizes between the 360°VR (number of fixations: 3.67 ± 0.57; number of saccades: 2.3 ± 0.34; search rate: 5.2 ± 2.5 *10^−4^) and 2D (number of fixations: 3.42 ± 0.66; number of saccades: 2.5 ± 0.63; search rate: 3.9 ± 1.9 *10^−4^) conditions (Figure 3). There was no significant main effect of Sex on any of the gaze variables (*p*s > 0.05).

### 3.2. Head excursion

**Figure 4:**
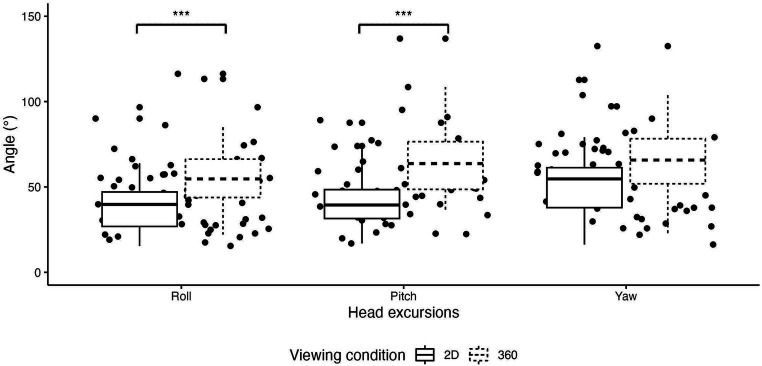
Box plots comparing head excursions in degrees between the two viewing conditions in Roll orientation, Pitch orientation, and Yaw orientation (***p < 0.001).

The linear mixed model revealed no significant interaction between Viewing condition and Sex on any head excursion variables (*p*s > 0.05). However, linear mixed model revealed small to medium significant main effect of Viewing condition on the roll (β = 42.79, 95% CI [31.09, 54.48], *p* < 0.001; std. beta = −0.44) and pitch (β = 20.11, 95% CI [8.14, 32.08], *p* = 0.001; std. beta = 0.78) axes. Indeed, participants had significantly higher number of rotations in the 3D-360°VR (respectively roll and pitch axes: 55.7 ± 22.3; 71.2 ± 35.1) compared to 2D (respectively roll and pitch axis: 39.9 ± 18.8; 41.8 ± 16.6) condition (*p*s < 0.001). The linear mixed model revealed no significant effects of the Viewing condition on the yaw axis (*p* = 0.98, d = 5.44e-03; Figure 4). There was no significant main effect of Sex on any head variables (*p*s > 0.05).

### 3.3. Simulation workload measures

**Figure 5:**
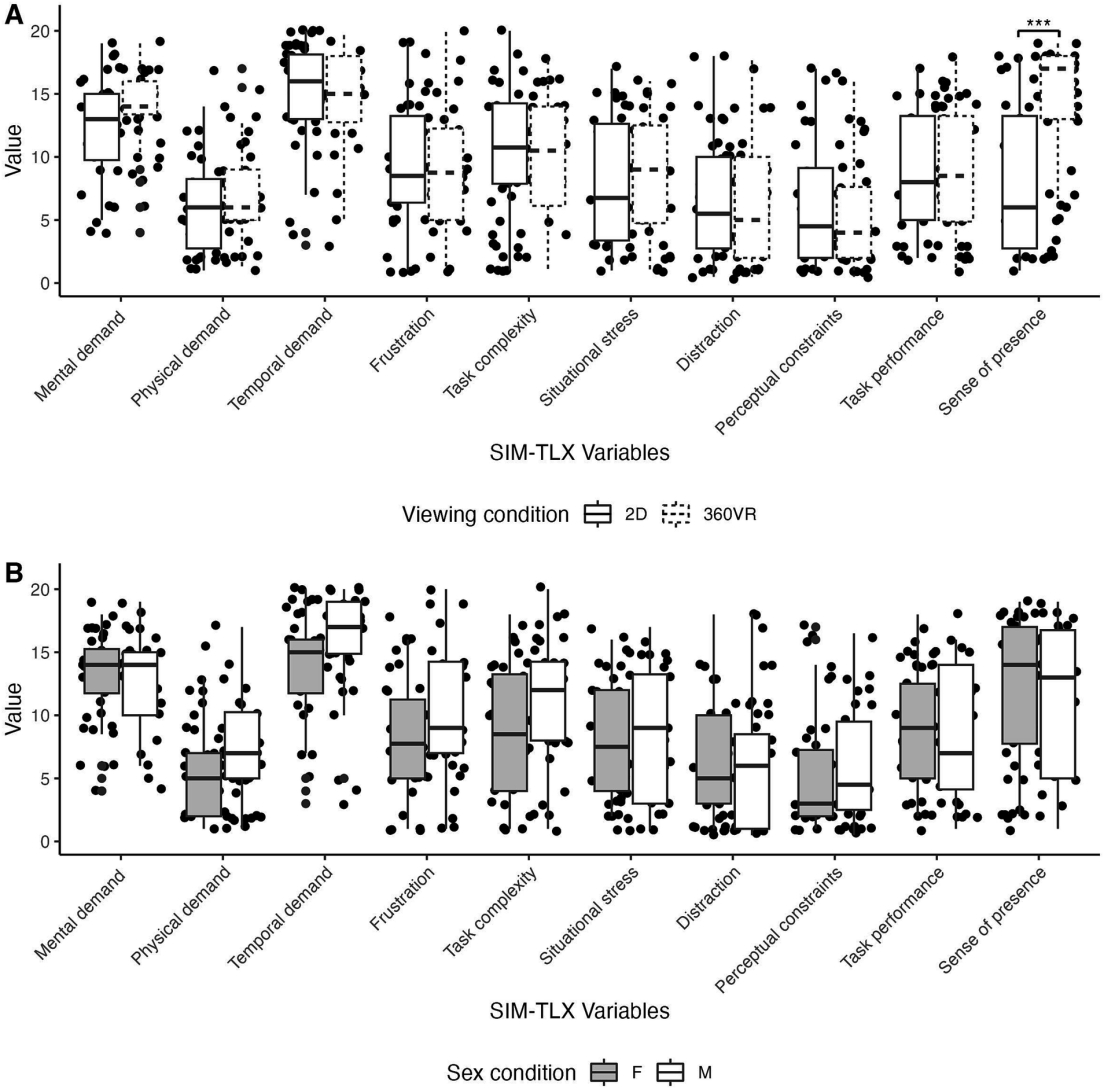
Box plots comparing **(A)** SIM-TLX variables between the two viewing conditions, and **(B)** SIM-TLX variables between the two sex conditions (***p < 0.001).

Linear mixed models revealed no significant interaction between Viewing condition and Sex for any of the SIM-TLX variables (*p*s > 0.05). However, linear mixed models revealed a large significant main effect of Viewing condition on the sense of presence item (β = 4.81, 95% CI [0.70, 8.92], *p* = 0.022; std. beta = 0.88). Participants reported a level of presence that was higher in the 3D-360°VR condition (15.1 ± 3.4) compared to the 2D condition (7.8 ± 5.4, *p* < 0.001). There was no other significant effect in any of the SIM-TLX items (*p*s > 0.05). There was no significant main effect of Sex on any of the SIM-TLX variables (*p*s > 0.05; Figure 5).

### 3.4. Heart rate

Linear mixed models revealed no significant interaction between Viewing condition and Sex on heart rate (*p* = 0.36). There was no significant main effect of Sex in either the 2D (females bpm: 96.6 ± 12.6; males bpm: 90.5 ± 11.0) or 3D-360°VR (females bpm: 95.1 ± 12.8; males bpm: 90.4 ± 9.3) condition. Similarly, there was no significant main effect of Viewing condition (2D bpm: 93.6 ± 12.1; 360°VR bpm: 92.8 ± 11.3).

## 4. Discussion

This study is the first to investigate the effects of 3D-360°VR versus 2D viewing conditions on gaze behavior, head excursion and workload during a boxing-specific anticipatory task. Our findings indicate that the 3D-360°VR condition resulted in shorter fixation duration and mostly greater head excursion behaviors compared to the 2D condition, partially supporting our first hypothesis (only H1b). Additionally, the analysis of simulation workload revealed a sense of presence that was much higher in the 3D-360°VR condition, highlighting its immersive effect (H2b). No other differences were observed in workload or external load between the two conditions, and there were no significant differences between sexes in this study.

### 4.1. Gaze behavior

The results show a shorter mean fixation duration in the 3D-360°VR, when compared to the 2D viewing condition, while the number of fixations and saccades, as well as the search rate, were not statistically different between conditions. These findings do not underlie drastically different gaze behaviors between conditions, but still seem to show that participants spent slightly less time fixating in the 3D-360°VR condition, during this anticipatory task. The 3D-360°VR condition provided more visual information due to its panoramic immersion compared to 2D, and participants may have spent less time on the same exact source of information. However, the results for saccades, number of fixations and search rate do not suggest that participants were exploring the visual scene to a greater extent. There are few comparable studies in the current literature. Nonetheless, these results differ from those reported in one study comparing VR, 3D and 2D conditions during category learning tasks (Barrett et al., 2022). In their study, the authors found more fixations of longer duration in the VR compared to the 2D condition. However, their experimental design involved several learning tasks in the form of symbols and shapes which differed greatly from ours. These tasks were not anticipatory in nature, were non-dynamic and void of temporal pressure. These differences in task characteristics may explain why the viewing modes had different effects on gaze behavior compared to our study.

Importantly, the head excursion analysis revealed rotations of greater amplitude in the 3D-360°VR compared to the 2D viewing condition. These results suggest that, while participants did not differ much in their gaze behaviors, there was a tendency for increased exploration or reaction with head movements, in the 3D-360°VR condition. Our first interpretation is that the panoramic information provided by the 3D-360°VR condition led participants to rely more on head movements for searching, instead of relying on large saccades that may result in the loss of crucial information. For instance, we noticed that participants exhibited more pronounced forward and backward tilting movements (pitch) in the 3D-360°VR condition. This suggests that they were actively exploring the visual scene, presumably to visually integrate the entire body of the opponent, which is not required in the 2D condition. Indeed, in 2D, the visual information is limited to the angle FOV, compared with the FOV in the 3D-360°VR condition, allowing participants to effectively integrate the entire body of the opponent in their visual field. As a result, the reduced necessity for head exploration in the 2D condition aligns with the longer fixations observed, as the majority of the information is concentrated in the central position of the projection. Our second interpretation of the head excursion results complements the previous one and suggests that participants, due to the immersive conditions, exhibited more reactive movements in 3D-360°VR, resulting in larger amplitudes of roll. In other words, when anticipating punches, participants produced greater lateral avoidance movements with their heads (roll). Overall, these findings suggest that the 3D-360°VR condition led to execution of larger movements and favored perception-action coupling compared to the 2D condition, primarily due to its immersive properties. This suggests that 3D-360°VR is a technology that could enhance the action correspondence of video-based test and consequently improve its validity (Hadlow et al., 2018). However, more research is needed to confirm this causal relationship. With regard to left and right head turns (yaw), no difference was observed between conditions. This result was not unexpected considering that boxing compared to team sports for example, does not involve exploration of the environment (e.g., scanning).

More specifically, previous studies in combat sport experts have shown that elite boxers tend to focus their gaze on the head and torso of the opponent, employing a gaze anchoring strategy, while novices tend to concentrate more on the forehand and pelvis region of the opponent (Ripoll et al., 1995; Hausegger et al., 2019; Martínez de Quel and Bennett, 2019; Vater et al., 2020). These gaze anchors are important in boxing, because they are distance-optimized locations between relevant cues that have been found to assist athletes in perceiving the opponent’s body more comprehensively and detecting movement initiation without initiating saccades, thus aiding in concealing their intentions (Hausegger et al., 2019; Vater et al., 2020). Although we cannot assume that novice participants in our study adopted such anchoring strategies, it would be interesting to replicate the present experiment with elite boxers and incorporate an analysis of areas of interest to investigate the presence of gaze anchoring strategies. As such, it is important to acknowledge that the interpretation of our findings was limited, and it was not possible to determine visual and head strategies in details (e.g., areas of interest). Likewise, it was not possible to identify if the fixations were used by participants to extract direct information through central vision or served as gaze anchors from which peripheral information was gathered (e.g., covert attention; Vater et al., 2020), because of the inherent difficulty to evaluate where attention is focused.

It should also be noted that we cannot ruled out the possibility that the differences between the 2D and 3D-360°VR conditions came from disparities in the size of the field of view. While this study intended to replicate typical video-based tests such as those found on computer screens (covering about 30° of visual field), it could be interesting to test whether the differences observed in the present study persist between 2D and 3D-360°VR viewing conditions of similar FOV. Finally, it cannot be ruled out that the decreased fixation durations and increased head excursions could be attributed to a novelty effect due to the exposure to an immersive environment (Barrett et al., 2022). However, we believe that presenting both conditions in the VR headset, as well as including a familiarization phase, may have helped avoid such bias. Despite these limitations, we believe there is significant added value in continuing to evaluate the validity of such technology, particularly in a sport like boxing which presents a high risk of injury (Di Virgilio et al., 2019). For example, this kind of tool could be useful in quantifying the risk of repeated impacts (Parr et al., 2023), or in proposing complementary and safe training tools that limit the impact of repeated blows (Lozano-Tarazona and Mauricio Rivera Pinzón, 2023), such has previously demonstrated in soccer.

### 4.2. Simulation workload measures

Consistent with our second hypothesis (H2b), measures of simulation workload showed a much higher sense of presence in the 3D-360°VR compared to the 2D condition. This result was consistent with previous studies reporting similar levels of presence in participants using VR and 360°VR displays (Akbaş et al., 2019; Harris et al., 2022; Romeas et al., 2022). Notably, the sense of presence reported in the present study was higher (15.1 ± 3.4) compared to a previous case-study employing boxing-specific scenarios in 360°VR without stereoscopic features (9.17 ± 2.2; Romeas et al., 2022). The presence of stereoscopy might be a reason to explain such a difference, which would support the importance of binocular depth information for improving stimulus correspondence such as previously reported (Hohmann et al., 2016). Such features should then be recommended for improving stimulus correspondence and consequently, task validity (Hadlow et al., 2018).

No other items from the simulation workload questionnaire (SIM-TLX) significantly differed between both conditions. This was inconsistent with our second hypothesis (H2) supporting that VR would increase the general workload of the task. However, this result is not totally inconsistent with the existing literature on the effect of VR on workload. Indeed, previous studies have sometimes reported an increase (Makransky and Petersen, 2021; Barrett et al., 2022) and sometimes no effect (Chao et al., 2017) of VR on workload. Here, we suggest that the short duration of the boxing scenarios and the low demand imposed by the avoidance task may have contributed to the null effect between conditions. Previous research has also suggested that the fixed focus distance could explain possible workload differences experienced in VR (Turnbull and Phillips, 2017). However, in our study, the focus distance was kept constant across conditions. In fact, it is important to note that both conditions were presented in the VR headset, reinforcing the chances that the null-findings were not influenced by the hardware display but rather attributed to a non-significant impact of the viewing conditions (360°VR, 2D) on workload.

While the task itself had relatively low demands (e.g., avoiding punches), the sense of presence emerged as a notable distinguishing feature between the 360°VR and 2D conditions. In terms of external load, heart rate measures were similar between conditions, which was to be expected given the low task demand. However, this finding raises questions beyond the scope of this study, about the potential arousal effect generated by the immersion in 360°VR vs. 2D displays (Bird et al., 2021), and highlights the need for further studies exploring scenarios of longer duration and incorporating measures of heart rate variability (Malińska et al., 2015).

### 4.3. Strengths, weaknesses, and perspectives

This study presented a number of strengths and weaknesses. Among the strengths, the same device (VR headset) was used to display two different viewing conditions, which facilitated the experimental organization, the standardization of the conditions, and the familiarization of the participants with the two modes of presentation. Given the adequate sample size, based on a power analysis, and the equal distribution of female and male participants who were recruited, results of this study should be representative of healthy young adults and contribute to bridge the gap in the under representation of women in sport science. In addition, we used both objective (gaze behavior, heart rate) and subjective (questionnaire) measures to describe our findings. Finally, this study voluntarily focused on a novice population with no previous experience in boxing for two main reasons. First, visual strategies observed in novices should be purely attributed to a condition effect and not entailed by an effect of expertise. Second, novice population could be easier to recruit for this study, as a preliminary study.

One limitation of our study is that the use of 2D and 3D-360° videos restricted the analysis of certain gaze behaviors that can be more comprehensively studied in VR, such as areas of interest (Luke et al., 2022). Examining the specific locations participants were looking at in each condition would have provided a more detailed comparison. Previous research has demonstrated that experts in boxing (Martínez de Quel and Bennett, 2019) and combat sports (Zhang et al., 2022) utilize specific areas of interest during visual exploration. Experts tend to distribute their gaze more towards areas such as the head and trunk of the opponent, while novices tend to focus more on the forehand and pelvis regions. In addition, although we found differences in gaze behavior between 3D-360°VR and 2D conditions, it was not possible to conclude if the strategies used in 3D-360°VR were closely related to those that would be observed in naturalistic conditions. In that sense, a next step of this project would be to compare viewing conditions between 2D, 3D-360°VR, VR and the real world in expert boxers, as well as to include measures of task performance. This will be of high interest considering that previous evidence has found that sport-specific visual strategies that have been linked to performance execution (e.g., quiet eye) could be disrupted in VR compared to real-world conditions (Harris et al., 2020b). Importantly, videos used in future research or application should be both verified and evaluated by experts to confirm their representativeness, as stimulus correspondence is key.

## 5. Conclusion

In this study, the effect of two viewing conditions (3D-360°VR, 2D) on gaze behavior, head excursion and workload in a boxing-specific anticipatory task was compared. Significant differences were observed between the two viewing modes, with 3D-360°VR leading to shorter fixation durations and greater head excursions. It was also demonstrated that 3D-360°VR induced a greater immersion, with participants reporting a sense of presence that was two times higher in 3D-360°VR compared to 2D. These preliminary findings report differences between 3D-360°VR and 2D displays and suggest that gaze behavior, head excursion and presence are proxies that should be further explored to explain the possible advantages of 3D-360°VR. In addition, studies comparing this viewing mode to real-world conditions will be needed to validate that 3D-360°VR favors naturalistic visual strategies and is a relevant paradigm that could be put to the test in assessing and training perceptual-cognitive skills in sport.

## Data availability statement

The original contributions presented in the study are publicly available. This data and code can be found in https://github.com/INSQuebec/Boxing using a workflow for Open Reproducible Code in Science (WORCS) proposed by Van Lissa et al. (2021).

## Ethics statement

The studies involving humans were approved by Research Ethical Committee of École de technologie supérieure of Montreal (H20220103). The studies were conducted in accordance with the local legislation and institutional requirements. The participants provided their written informed consent to participate in this study.

## Author contributions

TR, DL, and ML contributed to conception, design of the study, manuscript revision, read, and approved the submitted version. LJ organized the database. ML performed the statistical analysis. LJ and ML wrote the first draft of the manuscript. All authors understand that they are accountable for all aspects of the work and ensure the accuracy or integrity of this manuscript.

## Funding

This research was funded by the Fédération Olympique de Boxe du Québec through the Programme de soutien au développement de l’excellence sportive from the Ministère de l’Éducation du Québec and the Mitacs Acceleration program.

## Conflict of interest

The authors declare that the research was conducted in the absence of any commercial or financial relationships that could be construed as a potential conflict of interest.

## Publisher’s note

All claims expressed in this article are solely those of the authors and do not necessarily represent those of their affiliated organizations, or those of the publisher, the editors and the reviewers. Any product that may be evaluated in this article, or claim that may be made by its manufacturer, is not guaranteed or endorsed by the publisher.
